# Public Knowledge about Dementia in Poland—A Survey Study

**DOI:** 10.3390/jcm12247675

**Published:** 2023-12-14

**Authors:** Alicja Skowronek, Katarzyna Bojkowska-Otrębska, Beata Łabuz-Roszak

**Affiliations:** 1 Student Scientific Association at the Department of Neurology, Institute of Medical Sciences, University of Opole, 45-052 Opole, Poland; skowronek.ala@gmail.com; 2 Clinical Department of Geriatrics, Stobrawskie Medical Center in Kup, 46-082 Kup, Poland; kbojkowska@poczta.fm; 3 Department of Neurology, St. Jadwiga Provincial Specialist Hospital, Institute of Medical Sciences, University of Opole, 45-052 Opole, Poland

**Keywords:** dementia, knowledge, risk factors, health education

## Abstract

Background: Based on worldwide estimates, the number of people with dementia will increase significantly in the coming decades. Therefore, knowledge about dementia and its modifiable risk factors plays a crucial role in prevention. Although dementia is still incurable, an early diagnosis might help to slow down its progression and improve the quality of patients’ lives. The aim of the study was to assess public knowledge about dementia and its risk factors in Poland. Methods: The research was conducted in 2022 using a self-constructed questionnaire by applying computer-assisted web interviewing (CAWI). Results: A total of 304 completed surveys were obtained (mean score of 16.95 ± 2.79 points out of 23.6). The scores were significantly higher for people associated with the medical community in comparison to those unrelated to the medical community (18.23 ± 2.61 and 16.15 ± 2.59, respectively; *p* = 0.0001). A moderate negative correlation was found between the results and the ages of the respondents (R = −0.44; *p* = 0.001). No statistically significant differences were reported in the results between people involved in providing care to patients with dementia or those who had a patient with dementia in the family and those who were not involved in such care or had no relative with dementia. Conclusions: Knowledge about dementia and its risk factors in Poland is not satisfactory and should be improved. Special attention should be paid to educating the families and caregivers of people with dementia.

## 1. Introduction

Although dementia is prevalent in the population for those over 60 years old, it is not a natural part of the aging process. Dementia is associated with progressive neurodegeneration and leads to the impairment of memory, speech and other cognitive functions, such as the ability to learn, count and understand and the ability to interpret spatial orientation. In the course of this disease, patients face many emotional disorders, including depression, irritability and agitation. Dementia also causes physical problems, such as balance and gait disorders, leading to falls and injuries, urinary and/or fecal incontinence or eating disorders. As a result, the progressive course of dementia results in a gradual limitation of independent functioning in daily activities and the loss of patient autonomy [1,2,3,4,5,6]. Alzheimer’s disease (AD) is the most common form of dementia that accounts for approximately 60–70% of all cases [6]. Dementia is the most frequent cause of disability and dependence on assistance in elderly populations in developed and developing countries [3,6].

Currently, approximately 55 million people worldwide are affected by dementia, and the number of cases is predicted to increase to 78 million in 2030 and 152 million in 2050 [1]. There were over 525,000 dementia patients in Poland in 2018, which accounted for 1.38% of the Polish population. Despite the decreasing overall population in Poland, it is expected that the number of patients suffering from dementia will exceed one million in 2050 (3.23% of the population). The main reasons for this trend are the demographic changes in Poland and the estimation that the number of people over 70 years in Polish society will double between 2018 and 2050 [7].

In 2019, dementia syndromes were the seventh most common cause of death due to non-communicable diseases worldwide [3]. Recent studies on the occurrence of dementia in various parts of the world have found that in highly developed countries, the number of age-related diseases is decreasing due to changes in health care, common access to education and lifestyle changes [8,9,10,11], while the number of cases related to obesity, diabetes and low physical activity is increasing [11,12,13]. In 2020, the Lancet Commission identified 12 modifiable risk factors of dementia that, altogether, could be responsible for up to 40% of all cases of dementia worldwide. These modifiable risk factors are as follows: low level of education, hypertension, hearing impairment not corrected with hearing aids, smoking, obesity, depression, physical inactivity, diabetes, limited social contacts, excessive alcohol consumption, intracranial injuries and air pollution. Changes in the above factors could potentially prevent or slow down the development of the disease. Although there are no clear data on the impact of diet on the development of neurodegenerative diseases, the World Health Organization (WHO) recommends the use of the Mediterranean diet [14]. Additionally, there are reports about the protective effect of vitamin D [15].

Currently, intensive research is underway on causal drugs for Alzheimer’s disease, and there are some drugs whose early implementation can slow down disease progression [6]. Considering the fact that dementia is an increasingly common health problem that has negative impacts on the lives of patients and their caregivers, it is extremely important for family doctors to recognize the symptoms of dementia at the earliest stage possible using the Clinical Dementia Rating (CDR) scale and to check whether the patient meets the DSM criteria. Despite the fact that screening tests assessing cognitive functions (such as Mini-Mental State Examination (MMSE)) are not recommended in asymptomatic patients, an additional diagnostic process should be performed immediately when alarming symptoms are noticed. They may include confusing appointment dates, forgetting to take regular medications, susceptibility to financial fraud and newly occurring behavior disorders, such as depression or significant anxiety. When such symptoms are noticed, objective psychological tests (e.g., the MMSE or Addenbrooke’s Cognitive Examination III (ACE-III)) and (if available) neuroimaging tests, particularly MRIs, should be performed [14,16,17]. The initiation of pharmacotherapy should be tailored to a patient’s expectations (if his or her cognitive abilities allow it) and to the family members who most often take care of the person with dementia [15].

At the same time, the role of non-pharmacological therapies should be emphasized. Such approaches include changes in modifiable risk factors and psychosocial interventions enabling patients and their caregivers to contact people who experience similar problems. Group exercises and physical and social skill training can significantly improve the quality of lives of patients and their families. Additionally, music therapy is reported to have a beneficial effect on brain functions by stimulating the areas responsible for speech, attention and memory. This type of therapy also appears to affect behavioral and emotional disorders that commonly occur and are difficult to treat in patients with dementia syndromes [16,17,18].

Widespread education about dementia and modifiable risk factors for neurodegenerative diseases should have a permanent place in society. Proper knowledge in this area may result in a partial reduction in morbidity and the earlier detection of dementia symptoms. Diagnosis at an early stage allows for better care for patients and their caregivers, and it enables the implementation of therapies that can slow down disease progression, postponing the loss of patient autonomy. Additional emphasis should be placed on educating caregivers so that they can better understand the disease.

In Poland, there are several programs providing knowledge concerning various health problems (e.g., stroke or hypertension), while there are none regarding dementia. In many other countries, such programs are widespread, and their introduction was preceded by a thorough assessment of knowledge in the respective society [19,20,21,22].

Therefore, the aim of this study was to determine the level of public knowledge about dementia in Poland. The proper assessment of the results could highlight knowledge deficits in the Polish society and emphasize the need for a national educational campaign. 

## 2. Materials and Methods

The study was conducted between March and May 2022. Computer-assisted web interviewing (CAWI) was used to collect data through an anonymous questionnaire created on the basis of the Google Forms program. The survey was made available on the social network and spread throughout Poland. The questionnaire in the Polish language was prepared specifically for the purposes of this study and was based on the Dementia Knowledge Assessment Scale (DKAS) [2]. It consisted of 21 questions, 18 of which were close-ended, single-choice questions (true/false) and 3 that were multiple-choice questions.

The task of the respondents was to answer non-scored questions related to age, gender, place of residence, relationship with the medical community, the presence of dementia in the family and experience in caring for a patient with dementia, followed by questions that verified knowledge about the symptoms of dementia and the factors increasing and reducing the risk of dementia. The respondents obtained 1 point for selecting the correct answer in a close-ended question, while in the case of the multiple-choice questions, they received 0.2 points for each correct answer and lost 0.2 points for an incorrect option. The maximum possible score was 23.6 points. After completing the survey, each participant had the opportunity to check the correct answers and read the explanations provided under the questions.

The consecutive sampling method was used, but only fully completed questionnaires were taken into account.

The data were processed using Microsoft Excel. The results were considered statistically significant if the *p*-values were ≤0.05. Measurable data were characterized using means (Xs) and standard deviations (SDs). Percentages were used for nominal data. The compliance of the distribution of variables with a normal distribution was verified using the Student’s *t*-test. The Pearson correlation coefficient was also used.

## 3. Results

A total of 304 respondents (236 women and 68 men) completed the entire questionnaire and were taken into the study. The mean age of the respondents was 34 years (SD = 12.82, from 17 to 76 years). More than half (59.87%) of the respondents had higher education, and 38.49% declared a connection with the medical environment.

Dementia was present in the family in 43.75% of respondents, while 71 people (23.36%) stated that they had cared for a patient with dementia in the past or were currently involved in providing such care. Eighteen (5.92%) respondents had never heard of dementia before.

The largest group of respondents was comprised of those residing in the Silesian Province (51.64%), while the least numerous group was comprised of residents in the Podlaskie Province (0.33%) (there were no participants from the Warmia-Masuria Province). Most respondents were city dwellers (88.15%). Detailed information related to the study group is provided in Table 1. 

The respondents scored an average of 16.95 ± 2.79 points (71.81%) on the questionnaire. The difference in the results obtained by women and men did not show statistical significance (17.09 ± 2.82 and 16.44 ± 2.63 points, respectively; *p* = 0.089). The respondents associated with the medical community scored a mean of 18.23 ± 2.61 points (77.23%), and the mean score for those who were not related to the medical community was 16.15 ± 2.59 (68.42%; *p* = 0.0001). A moderate negative correlation was found between the results and the age of the respondents (R = −0.44; *p* = 0.001). Neither the place of residence nor the education level correlated statistically significantly with the results (R = 0.1, *p* = 0.07; R = 0.04, *p* = 0.49, respectively).

Among the survey participants, higher scores were obtained by people who had heard or read about dementia syndromes before the survey compared to those who had never heard of dementia (17.13 ± 2.67 and 14.57 ± 3.66, respectively; *p* = 0.0001). A family history of dementia and having cared for a patient with dementia did not significantly influence the results. The above data are summarized in Table 2.

Despite the fact that most respondents (90.79%) answered the following question positively: “Have you ever heard or read about dementia/dementia syndromes?”, only 55.26% of the respondents knew that dementia is not a natural condition of the aging process. Most respondents (98.68%) were certain that dementia was the result of changes in the brain. Alzheimer’s disease was indicated as the most common form of dementia by 67.11% of the respondents, and 288 (94.74%) study participants thought that it was an incurable disease, 235 (77.30%) respondents believed that it occurred mainly in people over 60 years of age and 268 (88.16%) participants answered that dementia could occur in middle-aged patients. Most participants agreed that there were factors increasing (81.58%) and reducing (80.92%) the risk of developing dementia. Among the respondents, 92.43% reported that when in the company of a patient with advanced dementia, one should address him or her directly, 67.44% believed that the patient could contact others through body language, and nearly 28% of the respondents indicated that communication with such a patient was completely impossible. Of the study participants, 42.11% considered pharmacotherapy to be the best form of treatment for the disease and 90.79% noticed that people suffering from dementia usually had problems with making decisions. Most respondents (n = 266; 87.50%) correctly indicated that the symptoms of dementia did not appear suddenly. A majority of the study participants (n = 292; 96.05%) considered the statement “all forms of dementia are hereditary” to be false, and 286 (94.08%) respondents stated that the early diagnosis of dementia improved the quality of a patients’ life. A significant proportion of respondents (79.61%) indicated that the number of dementia cases in the world would increase. The answers to all close-ended questions are given in Table 3.

**Figure 1:**
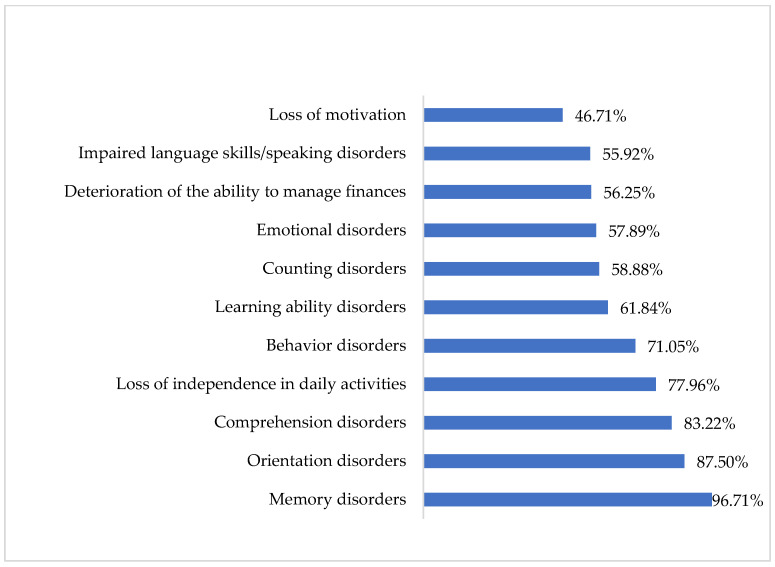
The symptoms of dementia indicated by the respondents (N = 304).

Among the symptoms typical of dementia, the respondents most often chose memory disorders (96.71%), orientation disorders (87.50%), comprehension disorders (83.22%), loss of independent functioning in everyday activities (77.96%) and behavioral disorders (71.05%). Loss of motivation was the least (46.71%) frequent symptom of dementia syndromes reported by the respondents. The mean number of selected symptoms was 7.54 ± 2.99. Only one symptom was indicated by five (1.64%) respondents, while all symptoms were selected by 96 (31.58%) participants (Figure 1).

**Figure 2:**
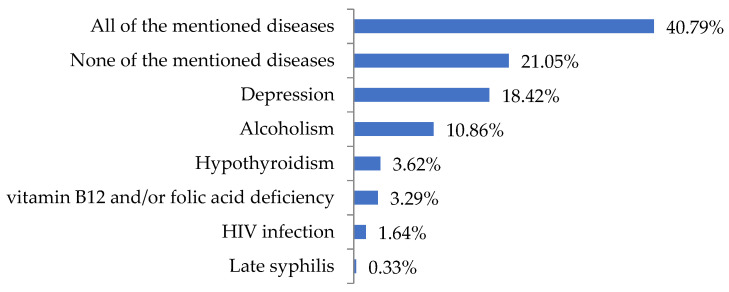
Reasons for dementia indicated by the respondents (N = 304).

Most respondents had some problems deciding whether dementia could be a symptom of the following diseases: depression, hypothyroidism, vitamin B12 and/or folic acid deficiency, late syphilis, HIV infection and alcoholism. Sixty-four (21.05%) respondents did not believe that dementia could be a symptom of the above diseases. One hundred and twenty-four (40.79%) respondents correctly indicated that dementia could occur in the course of other diseases (Figure 2).

**Figure 3:**
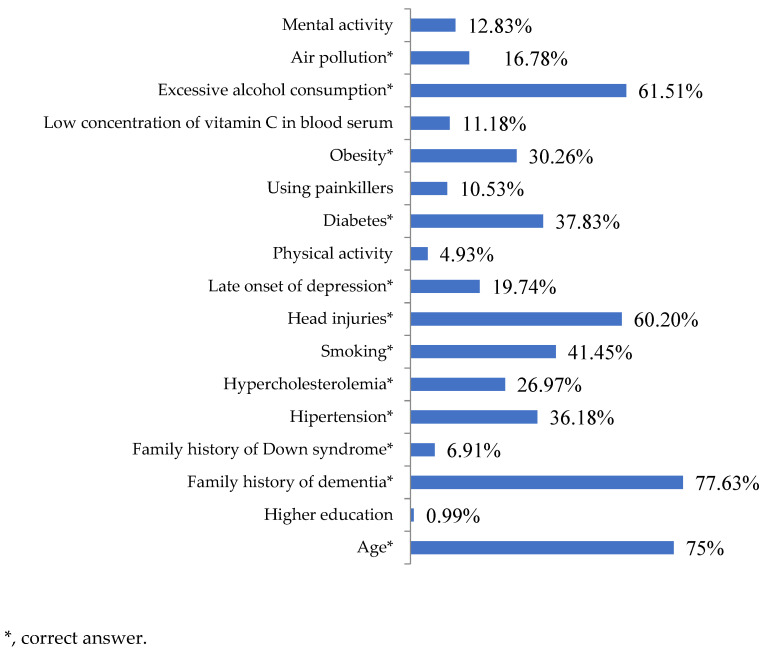
Factors increasing the risk of dementia according to the respondents (N = 304).

Most (81.58%) respondents correctly concluded that there were known factors increasing the risk of developing dementia. Among the given variants, the most frequently chosen were a family history of dementia (77.63%) and age (75%). Only 6.91% of the respondents noticed a connection between a family history of Down syndrome and an increased risk of dementia. Low serum vitamin C concentration and mental activity were incorrectly considered by some respondents to have an impact on the development of the disease (11.18% and 12.83%, respectively) (Figure 3).

**Figure 4:**
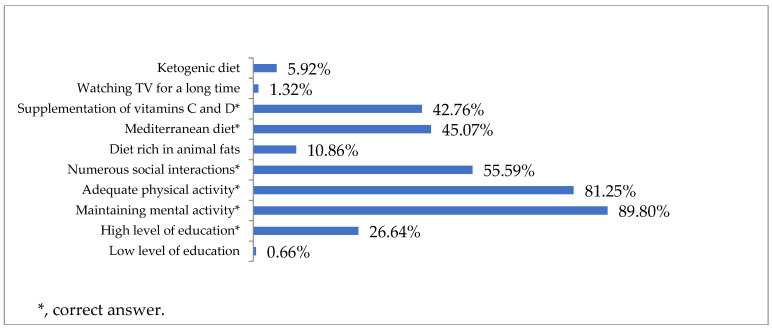
Factors reducing the risk of dementia according to the respondents (N = 304).

The respondents were less confident in indicating the existence of factors reducing the risk of developing dementia (80.92%). The most common preventive actions reported by the study participants included maintaining intellectual activity (89.80%) and physical activity (81.25%). Almost half of the respondents (45.07%) considered the Mediterranean diet to be beneficial for maintaining brain function, and 42.76% indicated that supplementation with vitamins C and D could have a protective effect (Figure 4).

## 4. Discussion

This study allowed the assessment of the knowledge of Polish society regarding dementia and the factors that could influence the development of the disease. To the best of our knowledge, this was the first such study in Poland, although Leszko et al. (2021) showed that the Polish version of the Alzheimer’s Disease Knowledge Scale (ADKS) could be a useful tool for assessing the effectiveness of educational programs aimed at caregivers, medical workers and the rest of the population [23]. However, so far, no studies have assessed the knowledge about dementia in Poland.

Based on the results, we could conclude that the knowledge about dementia syndromes is not sufficient among the Polish population. Similar conclusions could be drawn by analyzing the study results of Nielsen et al. (2016) among Danes and the immigrants from Poland, Turkey and Pakistan living in Denmark. Comparing their results with the findings of our survey, attention should be paid to the differences in the mean ages of the Polish respondents (our study: 34 ± 12.82 years; Dutch study: 65.1 ± 8.3 years) and the questionnaires used. The mean score for the Polish participants was 11.9 ± 2.6 points out of 19 (62.63%). Compared to the representatives of other ethnic groups, the Polish respondents obtained the highest number of points when answering questions related to the etiology of dementia as they most often indicated a family history of dementia (85%) and excessive alcohol intake (79%) as factors increasing the risk of developing the disease. The same answers were also among the most frequently chosen by the respondents in our study (77.63% and 61.51%, respectively). Danish researchers demonstrated significant differences in the level of knowledge about dementia among the representatives of selected ethnic groups, which appeared to be related to the differences in the levels of education obtained and acculturation [24].

Our study showed a moderate negative correlation between age and the points obtained in the survey, which was similar to a study conducted in South Korea [19]. However, such a finding did not correspond to the results obtained by Wu et al., who examined the knowledge of Chinese people about dementia [24]. The differences showed the necessity of adapting educational programs to the populations that underwent assessment. Wu et al. showed an interesting relationship between a higher level of knowledge and a less positive attitude toward dementia in older respondents compared to younger people characterized by a significantly lower level of knowledge related to dementia [24]. The importance of targeting elderly people during educational campaigns was identified by Smith et al., whose respondents aged 60 years and over considered dementia the third most important health issue associated with them [20].

In our study, significantly higher results were obtained by the group of respondents associated with the medical community (76.60% vs. 67.99%). However, these results are still not satisfactory, especially if we consider that the representatives of medical professions should be the first to provide reliable knowledge about dementia not only to the patients but also to their families and caregivers. Insufficient knowledge of medical staff has also been indicated in studies conducted in China and Australia [25,26,27,28,29,30], the analysis of which should encourage the introduction of additional educational programs on dementia syndromes among medical personnel worldwide. The differences in the level of knowledge of general practitioners working in different European countries were indicated by the significant disproportion in the percentages of diagnoses (40% of diagnoses by general practitioners in Germany [31] and 96% in Great Britain [32]). 

Of note, in our study, neither the people with a family member affected by dementia nor those involved in providing care to such patients had higher levels of knowledge than the other respondents (72% vs. 70.88% and 71.19% vs. 71.34%, respectively), which was not in line with the findings of Glynn et al., who observed Irish society [21]. People with a family member affected by dementia and those involved in providing care to such patients should be given adequate information about dementia. The lack of proper training and insufficient knowledge of caregivers of people with dementia result in lower quality in the services they provide and poorer comfort for patients and their families [33]. It appears that the insufficient knowledge of medical workers as well as their stigmatization of patients with dementia significantly increased stress among the families caring for patients. Caregivers expect greater support in coping with care activities, especially from community nurses [28,29]. Korean research showed that the higher the levels of knowledge and empathy of caregivers, the lower the caregiving burden they feel [34].

Because the factors that increase and reduce the risk of developing dementia are known, it is important to educate the public about them. In our survey, many (81.58%) respondents indicated the existence of factors increasing the risk of the disease, and 80.92% of respondents believed that there were protective factors. Knowledge in this area was also assessed in Norway, with 70% of responses indicating the presence of dementia risk factors [22], and in Germany, where the percentage of respondents demonstrating knowledge of the existence of modifiable dementia risk factors was 67.9% [35]. In the Polish, Norwegian and German populations, the two most frequently indicated factors that had a protective value against developing dementia were maintaining intellectual activity (89.80%, 84% and 86.7%, respectively) and physical activity (81.25%, 86% and 86.9%, respectively). A family history of dementia was indicated by 77.63% of our respondents and 58.1% of German respondents as a risk factor. Our respondents were less likely to associate smoking with an increased risk of dementia than Norwegians and Germans (41.45%, 53% and 53.5%, respectively). Poles, Norwegians and Germans were less likely to consider diabetes (37.83%, 26% and 38.2%, respectively), hypertension (36.18%, 31% and 36.1%, respectively) and being overweight (30.26%, 27% and 31.2%, respectively) to be risk factors for dementia. Only 16.78% of the respondents participating in our study considered air pollution a risk factor for dementia, while in the German study, 22.8% of respondents noticed the same relationship [34,35]. Insufficient public knowledge regarding the occurrence of risk factors was also reported in a study by Rosato et al. in Northern Ireland in which 56.4% of the participants incorrectly marked four or more dementia risk factors [36]. The analysis of the above data indicated a great need for public education in this area, with particular emphasis on the impact of modifiable factors on the occurrence of the disease.

Due to the urgent need for extensive education about dementia and its associated risk factors, studies have been conducted in many countries on the impact of courses on the initial and final level of knowledge of participants. The effectiveness of training provided as part of Massive Open Online Courses (MOOCs) was analyzed. Since 2013, the University of Tasmania has been conducting open, free and accessible courses entitled The Understanding Dementia Massive Open Online Course (UDMOOC) and the Preventing Dementia MOOC [37]. In 2016, 20,061 participants from 117 countries signed up to participate in the UDMOOC, and 29,039 participants from 132 countries participated in 2017. The analysis of the findings showed a significant increase in the level of knowledge after completing the course, regardless of the initial level of knowledge [38]. In Taiwan, the effectiveness of a 12-week mobile online course for caregivers of people with dementia was verified. It was shown that completing the training was associated with significant increases in the levels of knowledge of the participants. The effect was not only related to the period immediately after course completion but was also observed 12 weeks after completing the classes [39]. A completely innovative approach was to test the significant impact of short videos about dementia sent via WhatsApp in a population of elderly Chinese Americans. The study showed the possibility of using such applications as a promising educational form for the Chinese-speaking community [40].

Based on our data and the literature, there is an urgent need to create educational programs to expand society’s knowledge not only about dementia but also about the occurrence of (modifiable) risk factors. As our paper shows, various strategies are possible for reaching specific social groups, and appropriate media should be used for this purpose.

## 5. Limitation of the Study

It should be noted that despite our best efforts, our study had some limitations. The survey was prepared for the purposes of this study and was based on the DKAS translated into Polish, but the questionnaire was not validated. Since the survey was available only on a social networking site, some self-selection of the participants was possible. It was also difficult to reach the elderly due to their lesser activity on the Internet. Additionally, most respondents had higher education (59.87%) and secondary education (37.50%). Another limitation was the fact that the distribution of the respondents depending on the province was uneven (more than 50% were the residents of the Silesian Province). Moreover, the total number of people who started the questionnaire but did not finish it was unknown. 

## 6. Conclusions

We are the first to show that the knowledge of Polish people about dementia and its risk factors is insufficient. A negative correlation was found between the age and the results, which might indicate the need to develop educational programs precisely matched to a particular social group. 

People involved in healthcare showed statistically significant higher results than those who were not related to the medical community. However, these results were not satisfactory. It is crucial to increase the level of knowledge about dementia among doctors, nurses and other medical workers because medical staff should be a reliable source of knowledge for patients and their families.

Emphasis should be placed on educating the public about the risk factors for dementia. Through appropriate education of children, adolescents and adults, efforts should be made to minimize the stigmatization of people with dementia syndromes.

Particular attention should be paid to the education of the caregivers of patients with dementia, as our study showed that they did not have significantly greater knowledge than the other respondents. Moreover, it was demonstrated that greater awareness of caregivers regarding dementia syndromes reduced the burden of care for patients with dementia and had a positive effect on their attitude toward them. Public education regarding the risk factors and symptoms of dementia should become a priority of the national health policy.

## Figures and Tables

**Table 1:** Characteristics of the study group (N = 304).

**Mean Age, X ± SD**	34 ± 12.82
**Gender, n (%)**	
Women	236 (77.63)
Men	68 (22.37)
**Level of education, n (%)**	
Primary	2 (0.66)
Vocational	6 (1.97)
Secondary	114 (37.5)
Higher	182 (59.87)
**Place of residence, n (%)**	
Rural	36 (11.85)
Urban, city under 100,000 inhabitants	100 (32.89)
Urban, city from 100 to 500,000 inhabitants	110 (36.18)
Urban, city with over 500,000 inhabitants	58 (19.08)
**Province, n (%)**	
Lower Silesia	14 (4.6)
Kuyavian-Pomerania	3 (0.99)
Lodz Province	8 (2.63)
Lublin Province	10 (3.29)
Lubusz Province	5 (1.64)
Lesser Poland	19 (6.25)
Masovia	28 (9.21)
Opole Province	10 (3.29)
Subcarpathian Province	22 (7.24)
Podlasie Province	1 (0.33)
Pomerania	2 (0.66)
Silesia	157 (51.64)
Holly Cross Province	3 (0.99)
Greater Poland	14 (4.61)
West Pomeranian	8 (2.63)
Warmia-Masuria	0 (0.00)
**Do you study/work in the medical/medical-related industry?**	
Yes	117 (38.49)
No	187 (61.51)
**Have you ever heard or read about dementia/dementia syndromes?**	
Yes	276 (90.79)
No	18 (5.92)
I do not know	10 (3.29)
**Is there a history of dementia in your family?**	
Yes	133 (43.75)
No	128 (42.11)
I do not know	43 (14.14)
**Are you a person who has cared for or is currently caring for someone with dementia?**	
Yes	71 (23.36)
No	233 (76.64)

**Table 2:** The results for the different groups of respondents.

	% of Maximum Score (23.6 Points)	*p*-Value *
**Total**	71.81%	
**Gender**		
Women	72.54%	
Men	69.69%	0.089
**Do you study/work in the medical/medical-related industry?**		
Yes	76.60%	
No	67.99%	0.0001
**Have you ever heard or read about dementia/dementia syndromes?**		
Yes	72.06%	
No	61.25%	0.0001
**Is there a history of dementia in your family?**		
Yes	72.00%	
No	70.88%	0.429
**Are you a person who has cared for or is currently caring for someone with dementia?**		
Yes	71.19%	
No	71.34%	0.925

*, Student’s *t*-test.

**Table 3:** Answers to the close-ended questions (N = 304).

Question		Answers, n (%)	
		True	False
1	Dementia is a natural condition of the aging process.	136 (44.74)	* 168 (55.26)
2	Dementia is the result of changes in the brain.	* 300 (98.68)	4 (1.32)
3	The most common type of dementia is Alzheimer’s disease.	* 204 (67.11)	100 (32.89)
4	Dementia is curable in most cases.	16 (5.26)	* 288 (94.74)
5	Dementia mainly affects people over 60 years old.	* 235 (77.30)	69 (22.70)
6	It is possible to develop dementia in middle age.	* 268 (88.16)	36 (11.84)
7	There are known factors that increase the risk of developing dementia.	* 248 (81.58)	56 (18.42)
8	There are known factors that reduce the risk of developing dementia.	* 246 (80.92)	58 (19.08)
9	Communication with a person affected with advanced dementia is impossible.	85 (27.96)	* 219 (72.04)
10	People with advanced dementia often communicate through body language.	* 205 (67.43)	99 (32.57)
11	When in the company of a person with dementia, you should not address them directly because they will not understand what you are saying.	23 (7.57)	* 281 (92.43)
12	The best therapy for dementia is pharmacotherapy.	* 128 (42.11)	176 (57.89)
13	People with dementia usually have trouble making decisions.	* 276 (90.79)	28 (9.21)
14	The symptoms of dementia occur suddenly.	38 (12.5)	* 266 (87.50)
15	All forms of dementia are hereditary.	12 (3.95)	* 292 (96.05)
16	The early diagnosis of dementia improves the quality of a patients’ life.	* 286 (94.08)	18 (5.92)
17	The number of dementia cases worldwide will decrease in the coming years.	62 (20.39)	* 242 (79.61)

*, correct answer.

## Data Availability

The data presented in this study are available on request from the corresponding author.
